# Investigation of Discoloration of Anterior Teeth With Three Types of Substances Used in Endodontic Treatment

**DOI:** 10.1002/cre2.70204

**Published:** 2025-10-29

**Authors:** Sahar Soltani, Eshagh Ali Saberi, Nazanin Shahradnia, Pedram Abdollahzade Sangrodi, Elham Majidi

**Affiliations:** ^1^ Zahedan University of Medical Sciences Sistan and Baluchestan Province Iran; ^2^ Oral and Dental Disease Research Center Zahedan University of Medical Sciences Zahedan Iran; ^3^ Faculty of Dentistry, Deparment of Restorative and aesthetic dentistry, Student Research Committee Zahedan University of Medical Sciences Sistan and Baluchestan Province Iran

**Keywords:** discoloration, endodontic, endo‐materials, nanoparticles

## Abstract

**Objective:**

This study aims to investigate the discoloration of anterior teeth with three types of substances used in endodontic treatment.

**Materials and Methods:**

In this laboratory study, 75 newly extracted human anterior (central and lateral) teeth with full roots were examined. This study utilized the following materials: AH26, MTA Base (Maruchi), a novel formulation containing nano‐zinc particles, and a second novel formulation containing both nano‐zinc and nano‐copper particles. The teeth in the first three groups were dried with paper points, and the canals were completely sealed using the sealer lateral filling technique with gutta‐percha. Then the color of the samples was measured by a spectrophotometer at four times: initially, immediately after filling (0), 14 days, and 28 days later.

**Results:**

The results showed that the mean color change (∆E) at all times (immediately, 14 days later, and 28 days later) was statistically significant between all groups (*p* < 0.05). The degree of color change decreased in the following order: AH26 > Zn‐nano > Zn.Cu‐nano > novel nano‐zinc/copper material > MTA Base (Maruchi) > control group.

**Conclusion:**

The results of the present study showed that sealers with a zinc base (Zn‐nano and Zn.Cu‐nanoparticles) had an appropriate color change. Therefore, they can be used alongside other sealers available in the market.

## 1 Introduction

Tooth discoloration resulting from root canal treatment remains a challenging issue and many dental materials have the potential to cause internal tooth discoloration (Bosenbecker et al. 2020). These materials include endodontic sealers, retrograde and orthograde endodontic materials, amalgam, and resin‐modified glass ionomer (Goettems et al. 2020). Internal crown discoloration may exist before root canal treatment due to pulp bleeding, necrosis, dystrophic calcification after trauma, or aging. Additionally, antibiotic.corticosteroid materials used in the root canal can cause tooth discoloration, significantly affecting anterior teeth (Fagogeni et al. 2019; Tripathi et al. 2020).

Some specific elements like eugenol, phenol, and silver additives could lead to discoloration in the crown (Tripathi et al. 2020). Treating teeth that have been discolored due to dental procedures is harder, takes more time, and is less successful compared to teeth discolored from trauma (Noorsaeed et al. 2021). Consequently, to choose the best sealant, it is crucial for dental experts to have a deep understanding of how these materials can cause discoloration (Afkhami et al. 2019). Tooth discoloration due to sealers is caused by the penetration of their components into the dentinal tubules during or after sealer setting. The extent of sealer penetration and, consequently, the degree of tooth discoloration depend on factors like dentin thickness and sealer quality (Noorsaeed et al. 2021; Rozainah et al. 2020).

AH26 sealer creates chemical interactions in the root canal, resulting in the conversion of sealer components to bismuth, causing brown to green discoloration (Abu Zeid and Alnoury 2023). Silver corrosion in some sealers causes gray to black discoloration. Modified AHplus contains zirconium oxide for opacification, leading to long‐term tooth discoloration. Zinc oxide eugenol‐based and epoxy‐based sealers can also cause moderate to severe crown discoloration (Ashraf et al. 2020). The first MTA sealer produced was gray and could cause tooth discoloration. White MTA (WMTA) was developed to overcome this drawback (Ghabraei et al. 2024). The significant difference between WMTA and gray MTA is that WMTA contains fewer metal oxides like Al_2_O_3_, MgO, and FeO, which are the primary causes of their discoloration. However, WMTA has been shown to cause undesirable tooth discoloration. Additionally, MTA‐based sealers, despite their desirable properties like tissue compatibility and the ability to induce hard tissue formation, cause tooth discoloration (Ahmed and Abbott 2012; Jang et al. 2013; Belobrov and Parashos 2011).

As tooth color holds significant importance in dentistry, avoiding discoloration following root canal therapy, particularly in front teeth, has presented a clinical hurdle for dentists. This challenge demands considerable time to enhance the esthetics of discolored teeth. Despite continuous improvements, tooth discoloration following root canal treatment still exists (Bosenbecker et al. 2020; Moazzami et al. 2021; Ramos et al. 2016). Therefore, it is essential to evaluate materials for their potential to cause discoloration to approve them for use.

Despite ongoing advancements in endodontic materials, tooth discoloration following root canal treatment remains a significant clinical concern, particularly in anterior teeth where esthetics are of utmost importance. Several commonly used endodontic sealers, including AH26 and mineral trioxide aggregate (MTA)‐based formulations, have been associated with varying degrees of crown discoloration due to their chemical composition (Ahmed and Abbott 2012; Jang et al. 2013; Belobrov and Parashos 2011).

Recent developments in nanotechnology have introduced novel endodontic sealers incorporating nanoparticles such as zinc oxide (ZnO) and combinations of zinc and copper, which show promising physical and antimicrobial properties. However, no prior studies have specifically evaluated the discoloration potential of these newly developed nano‐zinc‐based and nano‐zinc. copper‐based sealers. This represents a critical gap in the current literature, especially given the increasing demand for biocompatible and esthetically favorable alternatives in endodontics. The clinical need for such materials is particularly relevant in the restoration of anterior teeth, where even minor discoloration can significantly compromise the overall esthetic outcome and patient satisfaction. Therefore, this study aims to investigate and compare the discoloration potential of two novel nano‐based sealers with conventional materials (AH26 and MTA Base), providing essential data for clinicians seeking optimal, esthetic solutions in endodontic therapy.

The null hypothesis of this study was that there is no significant difference in the amount of tooth discoloration, measured by color change (∆E), among anterior teeth filled with AH26, MTA base Maruchi, nano zinc particles, nano zinc and copper particles, or gutta‐percha alone (control) over a 28‐day period.

## 2 Methods and Materials

### 2.1 Study Group and Sample Size

This laboratory study was conducted on freshly extracted anterior teeth, from September 2022 to December 2022. After receiving ethics committee approval with the code IR.ZAUMS.REC.1399.550, sampling began. Inclusion criteria included freshly extracted, healthy anterior teeth extracted due to periodontal reasons from young individuals with periodontitis, teeth without discoloration, no cracks, no restorations, no fractures, and no caries. Exclusion criteria included any tooth damaged during the procedure.

Sample size was calculated using G*Power software (version 3.1.9.7) based on an a priori power analysis. An effect size (f) of 0.4 was assumed, corresponding to a medium effect according to Cohen's classification. With an α value of 0.05 and desired power of 80%, the analysis indicated a required sample size of 15 teeth per group. Considering a potential 10% loss due to technical errors or sample damage during processing, a total of 75 teeth were included across five groups.

### 2.2 Study Procedure

In this study, 75 freshly extracted human anterior teeth (central and lateral) with fully developed roots were used. Root canal treatment was performed immediately after extraction. The materials used in this study were AH26 (Dentsply DeTrey GmbH, Konstanz, Germany), MTA base Maruchi (a sealer from Korea with fine powder particles, pozzolan‐based MTA), a novel material containing nano zinc particles (ZnO nanoparticles), and a novel material containing nano zinc and copper particles. The teeth were stored in a sodium hypochlorite solution for 15 min to remove organic tissue residues, then washed with water, and the external root surface was debrided using an ultrasonic scaler. After preparing the access cavity, the working length was measured using a size 10 file, and the canals were prepared to the working length using rotary nickel‐titanium files (Dentsplay ProTaper, Switzerland) up to size F3, using sodium hypochlorite and 17% EDTA as irrigants.

### 2.3 Samples Classification

Seventy‐five teeth were divided into five groups (15 teeth per group): four experimental groups and one control group. The root canals in the first three experimental groups were dried using sterile paper points, and the canals were then obturated using the lateral compaction technique with standardized gutta‐percha cones and hand pluggers, in combination with the respective sealer. Excess sealer on the external root surface was carefully removed using cotton pellets moistened with normal saline. In the control group, gutta‐percha was inserted into the root canal without the use of any sealer.

Sealers were mixed according to manufacturers' instructions and applied using a lentulo spiral filler. Gutta‐percha was compacted laterally after sealing paste application.

### 2.4 Colorimetric Assessment

The color of the samples was measured by a spectrophotometer. The color of the samples was measured using a Minolta CM‐2600d spectrophotometer (Konica Minolta, Tokyo, Japan). Measurements were taken under standardized lighting conditions (D65 illuminant) and at a controlled room temperature of 23°C ± 1°C. Each measurement was repeated three times by the same calibrated observer to ensure intra‐examiner reliability, and the mean value was recorded. A black non‐reflective background was used during all assessments to maximize contrast and reduce environmental light interference.

The spectrophotometer was used to evaluate color matching at intervals immediately after filling (0), 14, and 28 days later. The samples were placed in the center of the device's aperture (6 mm in diameter), and direct light was shone on the surface of the samples, measuring the reflective spectrum according to the CIE Lab system. For all teeth, evaluation was performed using three parameters: L* (indicating value), a* (indicating the green‐red parameter), and b* (indicating the yellow‐blue parameter). The difference between tooth colors at initial and subsequent times was measured by ∆E, calculated as follows:

$$∆E=\left({\left(∆L\right)}^{2}+(∆{a)}^{2}+(∆{b)}^{2}\right)1^{1.2}$$

where ΔL, Δa, and Δb represent changes in L, a, and b from the initial time to subsequent times. All evaluations and measurements were performed by one observer using the device. ΔE₁ refers to color change from baseline to immediately post‐filling (within 1 h). ΔE₂ refers to the color change between immediate and 14 days. ΔE₃ refers to color change between 14 and 28 days.

### 2.5 Statistical Analysis

Statistical analysis was performed using SPSS software version 24 (SPSS Inc., Chicago, IL, USA). Chi‐square test was used to compare qualitative variables between groups. Kolmogorov‐Smirnov test was used to assess the normal distribution of all studied parameters. For normally distributed variables, repeated measures ANOVA was used. For non‐normally distributed variables, Kruskal‐Wallis test was used. Bonferroni post hoc test was used to compare means within each group at different times, and one‐way ANOVA and Bonferroni post hoc test were used to compare means between the five groups. A significance level of *p* < 0.05 was considered.

## 3 Results

In this study, 75 samples in five groups (MTA base Maruchi, AH26, Zn‐nano, Zn.Cu‐nanoparticles, and control) were examined. In the MTA base Maruchi group, novel material containing nano zinc, and novel material containing nano zinc and copper, the mean L* value showed significant statistical differences at all times (*p* < 0.05). Moreover, in the MTA baseS Maruchi group, AH26 group, Zn‐ nano, and Zn‐Cu nano, the mean a* value showed significant statistical differences at all times (*p* < 0.05). In the MTA base Maruchi group and novel material containing nano zinc and copper, the mean b* value showed significant statistical differences at all times (*p* < 0.05) (Table 1).

**Table 1:** The average characteristics of (L*), (a*), and (b*) color of extracted teeth before placement, immediately, 14 days and 28 days after placement of different sealers (*L, a*, and b* represent lightness, red‐green, and yellow‐blue chromaticity coordinates respectively, while ∆E represents the overall color difference from baseline at each time point).

Groups	Variables	Initial	immediately	14 days later	28 days later	*p*‐value
MTA‐ Maruchi	L*	71.06 ± 2.48	72.48 ± 2.23	72.67 ± 2.14	72.75 ± 2.54	< 0.0001
	a*	0.25 ± 0.14	1.06 ± 0.27	1.23 ± 0.24	1.20 ± 0.18	< 0.0001
	b*	17.26 ± 1.56	18.66 ± 1.69	18.71 ± 1.59	18.94 ± 1.87	< 0.0001
AH26	L*	67.34 ± 3.15	66.19 ± 5.47	66.03 ± 5.54	66.05 ± 5.94	0.192
	a*	1.56 ± 0.55	2.42 ± 0.94	2.49 ± 0.94	2.47 ± 0.97	< 0.0001
	b*	18.66 ± 3.05	18.32 ± 4.22	19.41 ± 2.85	19.13 ± 2.57	0.203
Zn‐nano	L*	68.42 ± 3.29	71.30 ± 3.22	70.81 ± 3.40	71.66 ± 3.31	< 0.0001
	a*	0.70 ± 0.34	1.69 ± 0.42	1.84 ± 0.34	1.87 ± 0.35	< 0.0001
	b*	20.18 ± 5.43	21.09 ± 4.07	21.31 ± 4.12	20.98 ± 3.79	0.167
Zn‐Cu nano	L*	67.97 ± 3.10	69.72 ± 3.47	70.00 ± 3.57	70.07 ± 3.55	< 0.0001
	a*	0.55 ± 0.35	1.30 ± 0.65	1.51 ± 0.58	1.56 ± 0.60	< 0.0001
	b*	16.04 ± 2.10	17.23 ± 2.59	17.30 ± 2.71	17.54 ± 2.87	0.007

The highest mean ∆E_1_ was observed in the AH26 group, followed by the Zn‐nano, Zn‐Cu nano, MTA base Maruchi, and control groups. Significant statistical differences were found in mean ∆E_1_ between the study groups (*p* < 0.05). The highest mean ∆E_2_ was observed in the AH26 group, followed by the Zn‐nano, Zn‐Cu nano, MTA base Maruchi, and control groups. Significant statistical differences were found in mean ∆E_2_ between the study groups (*p* < 0.05). The highest mean ∆E_3_ was observed in the AH26 group, followed by the Zn‐nano, Zn‐Cu nano, MTA base Maruchi, and control groups. Significant statistical differences were found in mean ∆E_3_ between the study groups (*p* < 0.05) (Table 2).

**Table 2:** Comparison of ∆E among different sealers during the investigated times (*ΔE₁ = baseline to immediate, ΔE₂ = immediate to 14 days, ΔE₃ = 14–28 days).

Groups	Immediately (∆E_1_)	14 days later (∆E_2_)	28 days later (∆E_3_)	*p*‐value
MTA base Maruchi	2.20 ± 0.46	2.43 ± 0.42	2.63 ± 0.71	0.003
AH26	4.13 ± 1.27	4.09 ± 1.13	4.55 ± 0.84	0.009
Zn‐nano	3.83 ± 0.95	3.83 ± 1.07	4.40 ± 1.12	< 0.0001
Zn‐Cu nano	3.01 ± 0.56	3.12 ± 0.79	3.29 ± 0.93	0.10
Control	0.86 ± 0.48	1.22 ± 0.38	1.17 ± 0.33	0.007
Total	2.80 ± 1.43	2.94 ± 1.31	3.21 ± 1.49	
*p*‐value	< 0.0001	< 0.0001	< 0.0001	

## 4 Discussion

Assessing the potential for discoloration in materials used in endodontic treatment is essential. The present study aimed to compare tooth discoloration using AH26 sealer, MTA base Maruchi, Zn‐nano and Zn‐Cu nano, using a spectrophotometer to reduce discoloration caused by endodontic treatment. The results showed that significant statistical differences in mean L*, a*, and b* values existed in the MTA base Maruchi and Zn‐Cu nano groups at all times. In the AH26 group, only a* values showed significant differences, while in the Zn nano, significant differences were observed in L* and a* values. Based on the statistical analysis, the null hypothesis was rejected, as significant differences in tooth discoloration (∆E) were found among the different endodontic sealers tested (*p* < 0.05).

The lateral compaction technique was selected for this study due to its widespread clinical use and reproducibility in laboratory settings. This method allows for controlled placement and compaction of gutta‐percha and sealer within the root canal system, ensuring consistent obturation quality across samples. Although newer techniques such as thermoplasticized gutta‐percha may offer improved adaptation, lateral compaction remains a well‐established and commonly used method, particularly in educational and general dental practice settings. Therefore, it provides a suitable model for evaluating sealer‐related discoloration under standardized conditions.

Ioannidis et al. (2013a) reported significant statistical differences in a* and b* values for MTA‐based sealers, similar to the present study for MTA base Maruchi in a* and b* values, but different for L* values due to differing conditions and times. The present study showed significant differences in ∆E in the MTA base Maruchi group over time, aligning with studies by Moazzami et al. (2021), Ioannidis et al. (2013b), Ramos et al. (2016), and Kang et al. (2015).

Significant statistical differences were also observed in ∆E in the AH26 group at three times (immediately, 14 days, and 28 days later), consistent with Shahrami et al. (2011). However, Chakmakchi et al. (2020). reported no change in AH26, differing from the present study due to different evaluation times and devices. The present study showed significant differences in ∆E in the Zn nano, but not in the Zn‐Cu nano, likely due to copper particles blocking dentinal tubules and preventing discoloration factors from entering the tooth structure.

The present study showed significant statistical differences in ∆E_1_, ∆E_2_, and ∆E_3_ among all groups, with the highest ∆E in AH26, followed by Zn‐ nano, Zn‐Cu nano, MTA base Maruchi, and control groups. Jahromi et al. (2011) reported similar findings for AH26. Tukey tests showed ∆E in MTA base Maruchi was significantly lower than AH26 and Zn‐ nano and significantly higher than the control, but not different from Zn‐Cu nano. Xavier et al. (2017) found no significant difference between MTA and control groups, differing from the present study due to differing sample populations (bovine teeth) and control groups.

AH26 showed significantly higher ∆E than Zn‐Cu nano, MTA base Maruchi, and control, but not different from Zn‐ nano. Ekici et al. (2019). reported significantly higher ∆E in AH26 than MTA base Maruchi, supporting the present study. The present study showed significantly higher ∆E in Zn‐ nano than MTA base Maruchi and control, but not different from AH26, with ∆E1 and ∆E2 not differing from Zn‐Cu nano, while ∆E3 was significantly higher.

Moazzami et al. (2021) reported significantly higher ∆E in Nano Fast Cement (NFC) than the control, aligning with the present study's findings. The present study showed significantly lower ∆E in novel material containing nano zinc and copper than AH26 and higher than the control, with no difference from MTA base Maruchi. The present study showed significantly lower ∆E in the control than all study groups, consistent with Moazzami et al. Tour Savadkouhi et al. (2018). found no significant difference between AH26 and control, differing from the present study due to different sample sizes and equal sample sizes in the control and AH26 groups.

Parsons et al. (2001) and Shahrami et al. (2011) showed differing discoloration potentials over time, with the highest discoloration observed 3 months later, while the present study did not evaluate this time, but showed increasing discoloration over time, supporting the present study. The present study showed the highest discoloration in AH26, followed by nano‐based materials (novel material containing nano zinc and novel material containing nano zinc and copper), with the least discoloration in MTA base Maruchi and the control. The present study had a relatively suitable sample size, and human extracted teeth were used for precise comparison of tooth discoloration, consistent with previous studies (Kang et al. 2015; Akbari et al. 2012; Felman and Parashos 2013). Despite efforts to create clinical‐like conditions, laboratory studies have limitations, so applying these results clinically should be done cautiously.

To interpret the clinical relevance of the observed color changes in this study, it is essential to consider established thresholds for color difference (∆E) values. According to recent literature (La Rosa et al. 2020), a ∆E value of 1.2 is considered the threshold for perceptibility, meaning that differences below this value are generally not detectable by the human eye under normal lighting conditions. A higher threshold of ∆E = 2.7 is defined as the acceptability limit, beyond which color differences are not only perceivable but also deemed clinically unacceptable or esthetically objectionable. In our findings, several groups exceeded the acceptability threshold of ∆E = 2.7 at various time intervals. Specifically, the AH26 group showed the highest mean ∆E values across all time points, surpassing the 2.7 threshold immediately after filling and continuing to increase over the 28‐day period. This indicates that the discoloration caused by AH26 is both visually noticeable and clinically significant, particularly in anterior teeth where esthetic outcomes are critical.

The novel material containing nano zinc particles also demonstrated ∆E values above 2.7 at later time points, suggesting a moderate risk of clinically unacceptable discoloration. In contrast, the novel material containing nano zinc and copper particles, as well as MTA Base Maruchi, remained close to or below the acceptability threshold throughout the observation period, indicating their potential suitability for use in esthetically demanding areas. These results emphasize the importance of selecting endodontic sealers with minimal discoloration potential, especially when treating anterior teeth. Sealers that exceed the ∆E = 2.7 threshold may compromise long‐term esthetics and could necessitate additional bleaching or restorative procedures, increasing both treatment complexity and patient discomfort. By applying these standardized thresholds, our findings align with previous studies that have emphasized the need for objective evaluation of color change using clinically relevant benchmarks rather than purely statistical significance. Incorporating such thresholds enhances the translational value of in vitro studies like ours and supports evidence‐based decision‐making in clinical practice.

## 5 Clinical Implications, Limitations, and Novelty

The findings of this study carry important clinical implications, particularly for endodontic treatments involving anterior teeth where esthetic outcomes are critical. The relatively lower discoloration potential observed for MTA‐Maruchi and the Zn/Cu‐nano sealer suggests that these materials may be more suitable for use in esthetically sensitive areas compared to other tested sealers such as AH26. Their reduced tendency to induce tooth discoloration supports their potential application in clinical scenarios where long‐term color stability of the restored tooth is a priority.

However, certain methodological limitations should be acknowledged. As an in vitro study, it utilized extracted human teeth stored in saline, which, although representative of natural dental structure, does not fully replicate the dynamic oral environment. Factors such as salivary flow, thermal changes, microbial activity, and long‐term staining from dietary elements were not accounted for. Therefore, while the results provide valuable preliminary data, future in vivo studies with extended follow‐up periods are recommended to confirm these findings under real clinical conditions.

In terms of novelty, this study represents the first comparative evaluation of newly developed nano‐zinc‐based and nano‐zinc/copper‐based sealers in relation to tooth discoloration. These materials offer promising properties, including potential biocompatibility and improved esthetics. By introducing and assessing these novel formulations alongside established sealers, this study contributes new insights into the development of endodontic materials that balance functional performance with minimal esthetic compromise.

## 6 Conclusion

Nano‐based sealers (novel material containing nano zinc and novel material containing nano zinc and copper) showed acceptable discoloration, so if sealing properties are confirmed in future studies, they can be used alongside other sealers like AH26 and MTA base Maruchi. The present study showed the least discoloration with MTA base Maruchi, making it suitable for esthetic purposes. AH26 showed the highest discoloration, so caution is recommended when using AH26 in anterior teeth root canal treatment, and the remaining sealer in the pulp chamber should be carefully cleaned with alcohol to prevent discoloration.

## Author Contributions

**Data collection:** Sahar Soltani, Eshagh Ali Saberi, Nazanin Shahradnia. **Data analysis:** Eshagh Ali Saberi, Nazanin Shahradnia. **Manuscript revision:** Pedram Abdollahzade Sangrodi, Nazanin Shahradnia, Elham Majidi. **Manuscript writing:** Eshagh Ali Saberi, Nazanin Shahradnia. **Statistical analysis:** Elham Majidi.

## Conflicts of Interest

The authors declare no conflicts of interest.

## Data Availability

All data are presented in the manuscript.

## References

[cre270204-bib-0001] Abu Zeid, S. T. , and A. Alnoury . 2023. “Characterisation of the Bioactivity and the Solubility of a New Root Canal Sealer.” International Dental Journal 73, no. 5: 760–769. 37244780 10.1016/j.identj.2023.04.003 PMC10509446

[cre270204-bib-0002] Afkhami, F. , S. Elahy , A. M. Nahavandi , M. J. Kharazifard , and A. Sooratgar . 2019. “Discoloration of Teeth Due to Different Intracanal Medicaments.” Restorative Dentistry & Endodontics 44, no. 1: 10. 10.5395/rde.2019.44.e10 PMC6387892 30834232

[cre270204-bib-0003] Ahmed, H. M. A. , and P. V. Abbott . 2012. “Discolouration Potential of Endodontic Procedures and Materials: A Review.” International Endodontic Journal 45, no. 10: 883–897. 22621247 10.1111/j.1365-2591.2012.02071.x

[cre270204-bib-0004] Akbari, M. , A. Rouhani , S. Samiee , and H. Jafarzadeh . 2012. “Effect of Dentin Bonding Agent on the Prevention of Tooth Discoloration Produced by Mineral Trioxide Aggregate.” International Journal of Dentistry 2012, no. 1: 563203. 22114601 10.1155/2012/563203 PMC3216310

[cre270204-bib-0005] Ashraf, H. , N. Mortezapour , S. Jabari , S. Zadsirjan , and F. S. Tabatabai . 2020. “Evaluation of Chemical and Physical Properties of an Experimental Endodontic Sealer in Comparison With AH‐26 and AH‐Plus.” Iranian Endodontic Journal 15, no. 3: 183–187. 36703809 10.22037/iej.v15i3.29153 PMC9709854

[cre270204-bib-0006] Belobrov, I. , and P. Parashos . 2011. “Treatment of Tooth Discoloration After the Use of White Mineral Trioxide Aggregate.” Journal of Endodontics 37, no. 7: 1017–1020. 21689563 10.1016/j.joen.2011.04.003

[cre270204-bib-0007] Bosenbecker, J. , F. J. Barbon , N. de Souza Ferreira , R. D. Morgental , and N. Boscato . 2020. “Tooth Discoloration Caused by Endodontic Treatment: A Cross‐Sectional Study.” Journal of Esthetic and Restorative Dentistry 32, no. 6: 569–574. 32043755 10.1111/jerd.12572

[cre270204-bib-0008] Chakmakchi, M. , C. Rahiotis , N. Kerezoudis , and A. Kakaboura . 2020. “In Vitro Assessment of Coronal Discoloration From Endodontic Sealers.” Al‐Rafidain Dental Journal 20, no. 2: 177–182.

[cre270204-bib-0009] Ekici, M. A. , A. Ekici , T. Kaskatı , and B. Helvacıoğlu Kıvanç . 2019. “Tooth Crown Discoloration Induced by Endodontic Sealers: A 3‐year Ex Vivo Evaluation.” Clinical Oral Investigations 23: 2097–2102. 30259191 10.1007/s00784-018-2629-1

[cre270204-bib-0010] Fagogeni, I. , J. Metlerska , M. Lipski , T. Falgowski , G. Maciej , and A. Nowicka . 2019. “Materials Used in Regenerative Endodontic Procedures and Their Impact on Tooth Discoloration.” Journal of Oral Science 61, no. 3: 379–385. 31378754 10.2334/josnusd.18-0467

[cre270204-bib-0011] Felman, D. , and P. Parashos . 2013. “Coronal Tooth Discoloration and White Mineral Trioxide Aggregate.” Journal of Endodontics 39, no. 4: 484–487. 23522541 10.1016/j.joen.2012.11.053

[cre270204-bib-0012] Ghabraei, S. , H. Assadian , H. Razmi , et al. 2024. “Comparison of the Antibacterial Effect of AH26, Adseal and Beta RCS Root Canal Sealers Against *Enterococcus faecalis*, an In Vitro Study.” Frontiers in Dentistry 21: 11–22. 38571896 10.18502/fid.v21i5.14853 PMC10985511

[cre270204-bib-0013] Goettems, M. L. , L. B. Thurow , T. G. Noronha , et al. 2020. “Incidence and Prognosis of Crown Discoloration in Traumatized Primary Teeth: A Retrospective Cohort Study.” Dental Traumatology 36, no. 4: 393–399. 32064750 10.1111/edt.12552

[cre270204-bib-0014] Ioannidis, K. , I. Mistakidis , P. Beltes , and V. Karagiannis . 2013a. “Spectrophotometric Analysis of Crown Discoloration Induced by MTA‐ and ZnOE‐Based Sealers.” Journal of Applied Oral Science 21, no. 2: 138–144. 23739854 10.1590/1678-7757201302254 PMC3881871

[cre270204-bib-0015] Ioannidis, K. , I. Mistakidis , P. Beltes , and V. Karagiannis . 2013b. “Spectrophotometric Analysis of Coronal Discolouration Induced by Grey and White MTA.” International Endodontic Journal 46, no. 2: 137–144. 22823058 10.1111/j.1365-2591.2012.02098.x

[cre270204-bib-0016] Jahromi, M. Z. , A. A. Navabi , and M. Ekhtiari . 2011. “Comparing Coronal Discoloration Between AH26 and ZOE Sealers.” Iranian Endodontic Journal 6, no. 4: 146. 23130069 PMC3471592

[cre270204-bib-0017] Jang, J.‐H. , M. Kang , S. Ahn , et al. 2013. “Tooth Discoloration After the Use of New Pozzolan Cement (Endocem) and Mineral Trioxide Aggregate and the Effects of Internal Bleaching.” Journal of Endodontics 39, no. 12: 1598–1602. 24238455 10.1016/j.joen.2013.08.035

[cre270204-bib-0018] Kang, S.‐H. , Y.‐S. Shin , H.‐S. Lee , et al. 2015. “Color Changes of Teeth After Treatment With Various Mineral Trioxide Aggregate‐Based Materials: An Ex Vivo Study.” Journal of Endodontics 41, no. 5: 737–741. 25732402 10.1016/j.joen.2015.01.019

[cre270204-bib-0019] La Rosa, G. R. M. , S. Pasquale , E. Pedullà , F. Palermo , E. Rapisarda , and A. M. Gueli . 2020. “Colorimetric Study About the Stratification's Effect on Colour Perception of Resin Composites.” Odontology/the Society of the Nippon Dental University 108, no. 3: 479–485. 10.1007/s10266-019-00469-9 31664633

[cre270204-bib-0020] Moazzami, F. , S. Sahebi , S. Shirzadi , and N. Azadeh . 2021. “Comparative In Vitro Assessment of Tooth Color Change Under the Influence of Nano Fast Cement and MTA.” Journal of Dentistry (Shiraz, Iran) 22, no. 1: 48–52. 33718530 10.30476/DENTJODS.2020.85057.1113 PMC7921768

[cre270204-bib-0021] Noorsaeed, A. S. , S. A. M. Lanqa , R. A. Alshehri , et al. 2021. “Causes and Management of Tooth Discoloration: A Review.” Journal of Pharmaceutical Research International 33, no. 60B: 969–976.

[cre270204-bib-0022] Parsons, J. , R. Walton , and L. Rickswilliamson . 2001. “In Vitro Longitudinal Assessment of Coronal Discoloration From Endodontic Sealers.” Journal of Endodontics 27, no. 11: 699–702. 11716085 10.1097/00004770-200111000-00012

[cre270204-bib-0023] Ramos, J. C. , P. J. Palma , R. Nascimento , et al. 2016. “1‐year In Vitro Evaluation of Tooth Discoloration Induced by 2 Calcium Silicate–Based Cements.” Journal of Endodontics 42, no. 9: 1403–1407. 27497511 10.1016/j.joen.2016.06.012

[cre270204-bib-0024] Rozainah, N. A. G. N. , A. N. Farah , and M. I. Karobari . 2020. “Management of Discolored Failure Root Canal‐Treated Upper Lateral Incisor.” Case Reports in Dentistry 2020, no. 1: 1–6. 10.1155/2020/8202873 PMC7273397 32547794

[cre270204-bib-0025] Shahrami, F. , M. Zaree , A. P. B. Mir , M. Abdollahi‐Armani , and A. Mesgarani . 2011. “Comparison of Tooth Crown Discoloration With Epiphany and AH26 Sealer in Terms of Chroma and Value: An In Vitro Study.” Brazilian Journal of Oral Sciences 10, no. 3: 171–174.

[cre270204-bib-0026] Tour Savadkouhi, S. 2018. “Ex Vivo Comparison of the Discoloration Potential of Two Endodontic Sealers in Human Incisors.” Journal of Islamic Dental Association of Iran 30, no. 1: 15–20.

[cre270204-bib-0027] Tripathi, R. , S. Cohen , and N. Khanduri . 2020. “Coronal Tooth Discoloration After the Use of White Mineral Trioxide Aggregate.” Clinical, Cosmetic and Investigational Dentistry 12: 409–414. 33061653 10.2147/CCIDE.S266049 PMC7533229

[cre270204-bib-0028] Xavier, S. R. , K. J. Pilownic , A. H. Gastmann , M. S. Echeverria , A. R. Romano , and F. Geraldo Pappen . 2017. “Bovine Tooth Discoloration Induced by Endodontic Filling Materials for Primary Teeth.” International Journal of Dentistry 2017, no. 1: 7401962. 28479918 10.1155/2017/7401962 PMC5396471

